# Sulfation pathways in times of change

**DOI:** 10.1042/EBC20230099

**Published:** 2024-12-04

**Authors:** Jonathan Wolf Mueller, Daniela Fietz, Irundika H.K. Dias

**Affiliations:** 1 Metabolism and Systems Science, College of Medicine and Health, University of Birmingham, Birmingham, U.K.; 2 Institute for Veterinary Anatomy, Histology and Embryology, Justus Liebig University, Giessen, Germany; 3 Aston Medical School, College of Health and Life Sciences, Aston University, Birmingham, U.K.

**Keywords:** conjugate analytics, pathways analysis, sulfate activation

## Abstract

Sulfation pathways are an essential part of overall sulfur metabolism. Sulfation pathways are mainly about sulfate activation, and the making and breaking of biological sulfate esters. This special issue features some extended reflection on what was presented at the SUPA 2023 meeting on Sulfation Pathways. Novel insights into the synthesis and analytics of sulfate, of sulfated conjugates, and of protein persulfides are presented. Oxysterol sulfates are presented as emerging sulfo-metabolites. Sulfation pathways enzymes are discussed in various disease settings. This special issue also presents insights into polysaccharide sulfotransferases and their functional characterization. Finally, cytoplasmic sulfotransferases are highlighted with regards to their impact on DNA-modification, and in the context of endocrine disruptors. All in all, thought-provoking findings, with the potential to guide and stimulate future research in the field of sulfation pathways and beyond.

Sulfation pathways are an essential part of overall sulfur metabolism. Sulfation pathways are mainly about sulfate activation, and the making and breaking of biological sulfate esters [1]. In human physiology, sulfation pathways are probably best known from conjugation reactions in the first-pass metabolism of the liver. During phase-II biotransformation, liver cells sulfate anything suitable that comes along, be it natural compounds, endogenous hormones, or drugs and other xenobiotics [2]. The field of sulfation pathways research is growing. Prominent sulfo-metabolites in focus include sulfated steroids [3] and sterols [4], sulfated vitamin D [5], or indoxyl sulfate [6]. The methods spectrum to study sulfation pathways is broad, ranging from biophysical [7] and computational approaches [8], via analytical and chemical methods [9–11], to rare-disease and clinical settings. Insights around sulfated proteoglycans, input from the *matrix* field, and exciting new chemo-synthetic routes towards sulfo-conjugates and their analytics, add to this list.

The SUPA 2023 meeting on Sulfation Pathways took place 12–14 September 2023 in Birmingham, UK, Figure 1. With about 30 participants, a rather small audience, the meeting offered ideal networking opportunities to our delegates. The audience was extremely diverse with people from Australia, Czechia, China, England, France, Germany, the Netherlands, Portugal, Saudi Arabia, Scotland, Slovenia, Turkey, the USA, and Wales. At SUPA 2023, the prize for the best talk, selected from abstracts, was given to Abdul Aziz Mhannayeh for their talk on ‘Redox regulation of PAPS synthase enzymes’. The SUPA-specific prize for ‘the longest sulfation pathway’ was awarded to Yugang Wang for their talk ‘The first glance of histone sulfation’.

**Figure 1:**
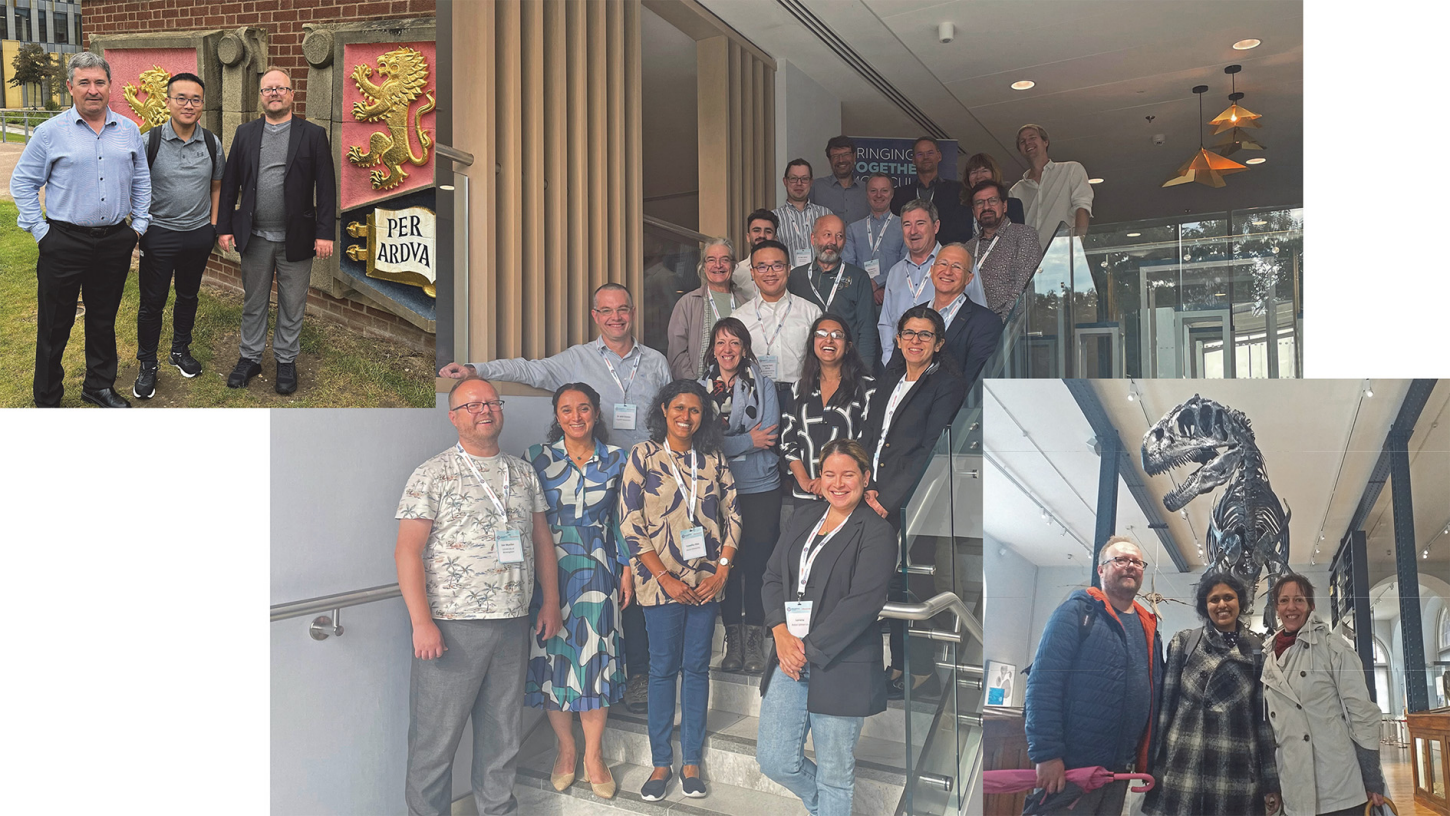
Impressions from the last meeting on sulfation pathways. SUPA 2023 took place in Birmingham, UK. In the aftermath to the conference, we offered campus tours, both at Aston University and at the University of Birmingham.

This special issue features some extended reflection on what was presented at the SUPA 2023 meeting. Paul Dawson made a very good case about the importance of the oxy-anion sulfate [12], arguing that we should spend more effort on quantitating sulfate in plasma and serum, both in research and in clinical settings. His case was seconded by Emil den Bakker and colleagues, who argued that sulfate would be a neglected, but highly relevant anion [13]. Ana Reis and Iru Dias then argue that oxysterol sulfates, made endogenously or from dietary intake, are emerging sulfo-metabolites [14]. To study the clinical significance, biological relevance and biophysical implication of oxysterol sulfates, they review analytical procedures to measure these sulfo-metabolites in fluids, cells, and tissues [14]. Tea Rizner showcased steroid sulfotransferase activity and how steroid sulfatase action modulates androgen and estrogen activity in gynecological cancers [15]. Same enzyme, completely different setting – Will Davies explored cardiac arrhythmia in individuals with steroid sulfatase deficiency [16]. Looking at Golgi-residing sulfation enzymes, Dave Fernig and Dulce Papy-Garcia discuss the genetic variability of putative sequences of polysaccharide sulfotransferases and their functional characterization [17, 18].

Thiophilic chemist Alan Jones discussed current and future approaches to the sulfation of small molecules [19], including essential approaches to obtain necessary reference material and standards. Within the wider sulfation pathways field, Nik Morton highlights analytical challenges around the quantification of persulfidation of specific proteins [20]. Another analytical challenge is detecting and quantifying interactions of proteins with heparan sulfate that Dave Fernig highlights [21]. More applied aspects are covered by Paul Foster highlighting steroid sulfation and desulfation in normal and malignant ovaries [22]. Jon Mueller, together with colleagues, reviews current literature around linking sulfation pathways and diabetes [23, 24]. Hansruedi Glatt reviews studies on the impact of sulfotransferase SULT1A1 on various substances to modify DNA. Finally, Michael Duffel provided insights about cytosolic sulfotransferases and their impact on, and inhibition by endocrine disruptors [25].

In the tradition of other sulfur meetings, SUPA 2023 ended with a scheduled hour of plenary discussion. Joint efforts in analytical challenges around sulfate quantification were encouraged. There was agreement that cross-sectional sulfation meetings, bringing together biomedical and plant researchers, are exceptionally useful and insightful. Finally, we discussed future meetings; how to retain the level of engagement and to aim for higher participant numbers. SUPA 2023 was the fourth SUPA meeting in a comparable setup. Previous meetings took place in Greifswald, Germany, 2015; 2017 we met in Birmingham, UK; and SUPA 2019 led us to Castle Rauischholzhausen, close to Giessen, Germany. All SUPA meetings were loosely associated with dedicated special issues or research topics in 2016 [26], 2018 [27], and 2022 [28].

We hope you enjoy this Special Issue with its bespoke cover. You can see tiles that each are tiny snapshots of scientific illustrations from the published authors of this special issue. Artist John Gage has abstracted signs, patterns, and symbols, leaning from Art Deco. The tiles then clad a wall from a virtual room; the orange diagonal might even remind us of some positive correlation. For this CoverArt, Jon and John describe their collaboration for this genuine Science and Art project elsewhere [29].

What is it that the future might hold? Maybe it is surprise and change. We can only recommend reading a recent study by Yugang Leo Wang reporting on the tyrosine sulfation of a histone protein by a cytoplasmic sulfotransferase [30]; something about a new structural conformation of steroid sulfatase [31]; or about new findings by Kurogi and colleagues [32]. They report about apparently ‘normal’ sulfotransferases that use the otherwise classic sulfate donor PAPS, for a non-classical addition of the sulfuryl moiety into an en-carbonyl acceptors, resulting in a true sulfonate with a carbon-sulfur bond [32]. All these studies are just some examples of recent dogma-challenging reports in the field. May the future of research into sulfation pathways be bright.

## Abbreviations

PAPS: 3'-phospho-adenosine-5'-phospho-sulfate
SUPA: sulfation pathways
